# Supplementary material for: The relationship between genomic variation and genetic load: insights from small island populations
Source: Heredity (Edinb). 2026 Mar 28;135(5):332–41. doi: 10.1038/s41437-026-00835-8 (PMC13219662; doi:10.1038/s41437-026-00835-8)
Supplement: Supplementary file 1 — Supplementary Information [file 41437_2026_835_MOESM1_ESM.docx]

**Supplementary Information for**

The relationship between genomic variation and genetic load:

insights from small island populations

Maëva Gabrielli, Andrea Benazzo, Roberto Biello, Alessio Iannucci, Daniele Salvi, Gentile Francesco Ficetola, Claudio Ciofi, Emiliano Trucchi, Giorgio Bertorelle

Corresponding author: Maëva Gabrielli and Giorgio Bertorelle

Email: [maeva.gab@hotmail.fr](mailto:maeva.gab@hotmail.fr), ggb@unife.it

**This file includes:**

Figures S1 to S10

Tables S1 to S8


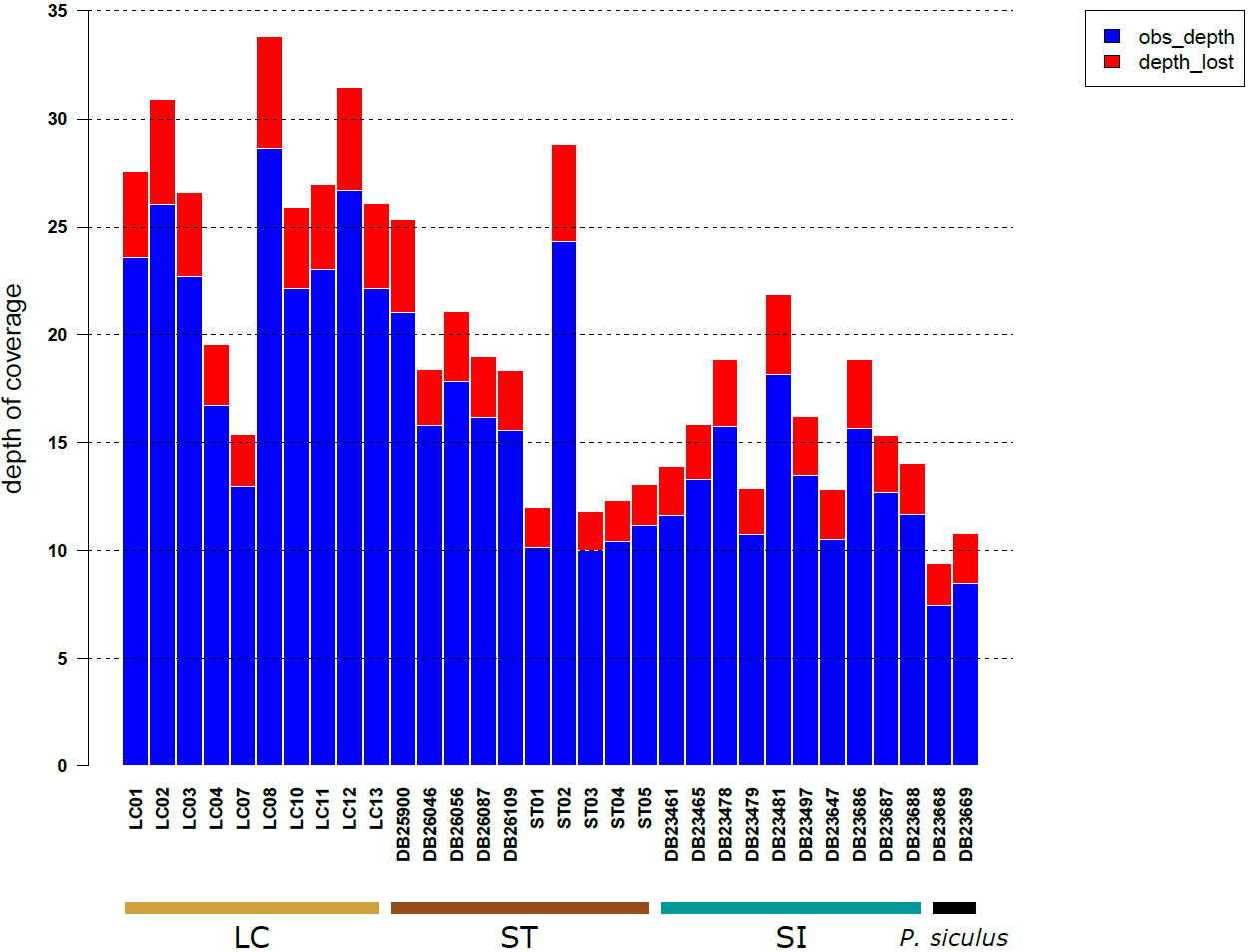


Fig. S1. Details about the individual depth of coverage of the 32 individuals sequenced for this study, for the Aeolian wall lizard (*P. raffonei*) from La Canna (LC) and Strombolicchio (ST), the Sicilian wall lizard (*P. waglerianus*, SI), and the Italian wall lizard (*Podarcis siculus*). The observed depth (in blue) is the depth after read mapping, while the depth lost (in red) represents the depth of coverage from the reads that did not map or did not pass the quality filters. The sum of the observed depth and depth lost is therefore the depth estimated from the initial number of reads.


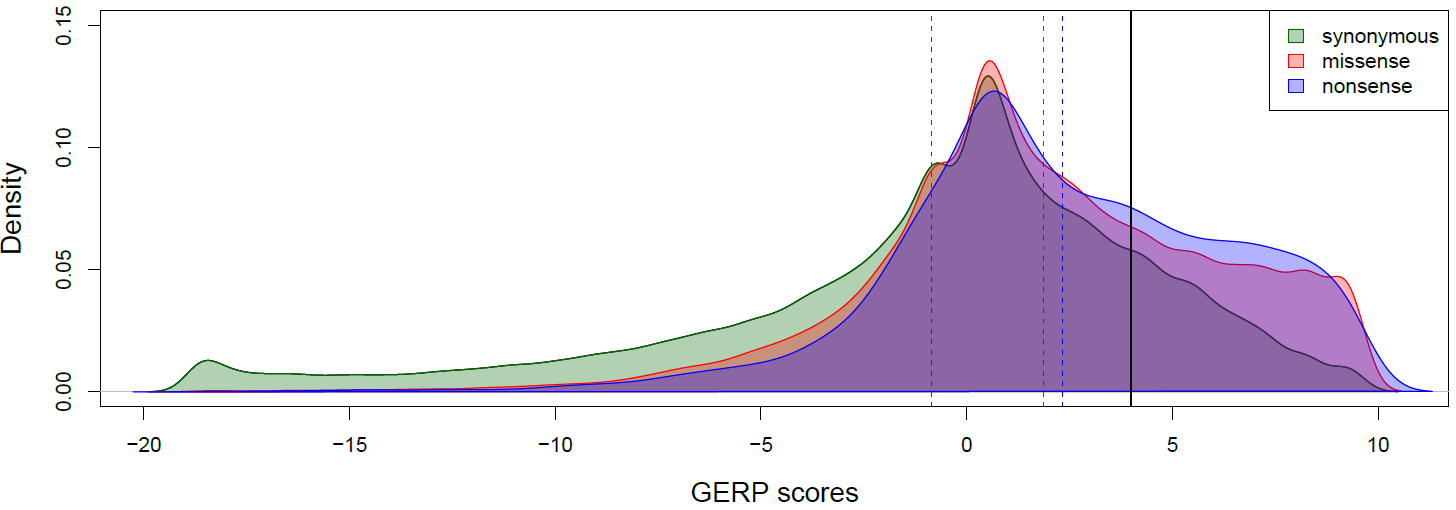


Fig. S2. Distribution of GERP scores for mutations identified as synonymous, missense and nonsense in SnpEff. Vertical dotted bars indicate the mean score for each type of mutations while the vertical plain bar indicate the hard threshold of GERP = 4. Nonsense and missense mutations have higher GERP scores than synonymous mutations, that in general have low GERP scores, as expected (significative differences between the mean score of synonymous and missense mutations: p-value < 2e-16; synonymous and nonsense mutations: p-value < 2e-16; and missense and nonsense mutations: p-value = 3.8e-13).


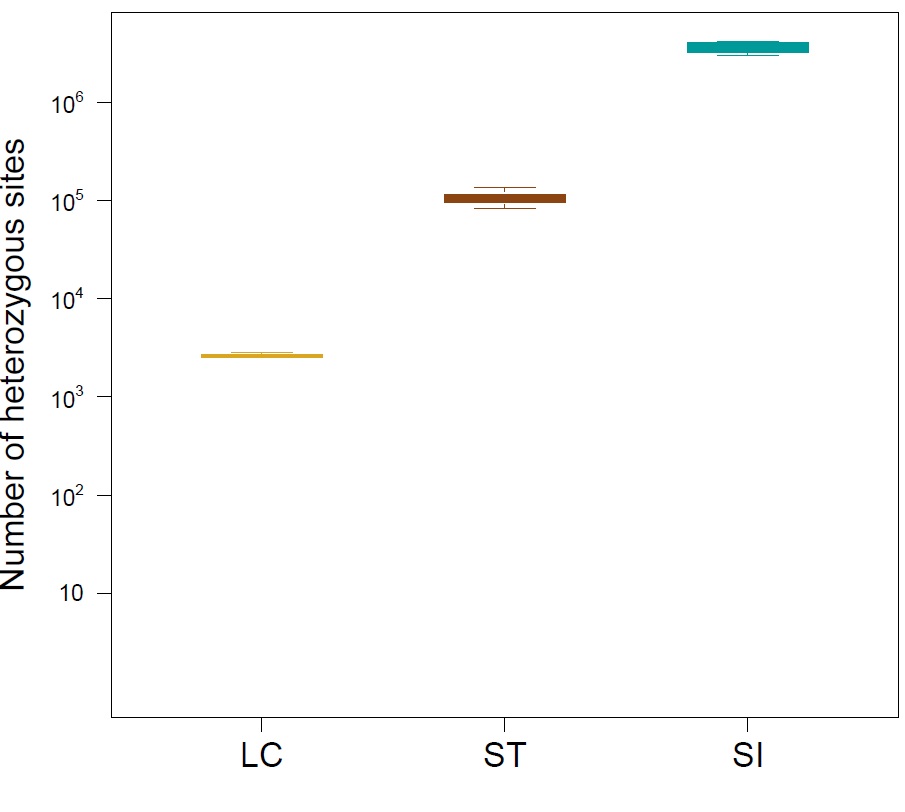


Fig. S3. Number of individual heterozygous sites for each group of interest: the Aeolian wall lizard from La Canna (LC) and Strombolicchio (ST), and the Sicilian wall lizard (SI).

**
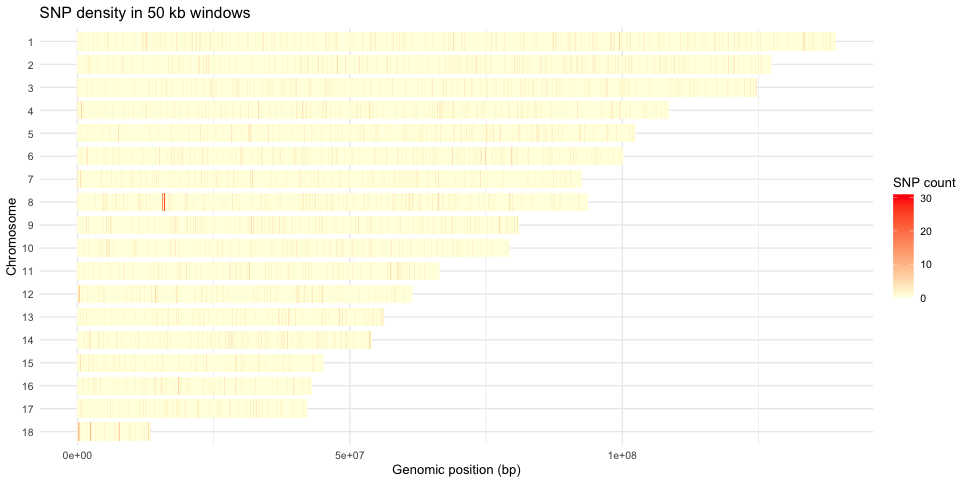
**

**Fig. S4.** SNP density in non-overlapping 50 kb windows in the 18 autosomes of La Canna population.


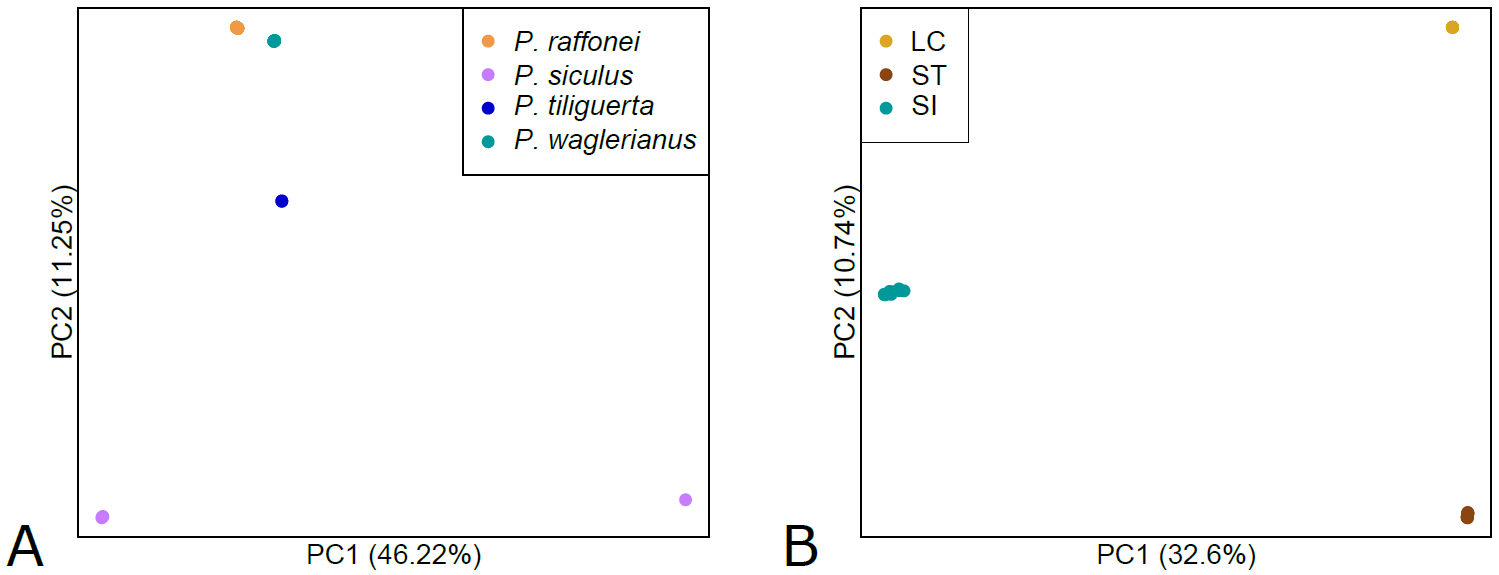


Fig. S5. Principal Component Analysis of the A 38 million of SNPs present in the 4-species dataset that includes 32 original genomes and 6 genomes from Yang et al. (2021) (the Aeolian wall lizard *P. raffonei* and the Sicilian wall lizard *P. waglerianus*, plus the outgroups *P. tiliguerta* and *P. siculus*) and the B 20 million of SNPs in our three groups of interest: Aeolian wall lizards from La Canna (LC) and Strombolicchio (ST), and Sicilian wall lizards (SI).


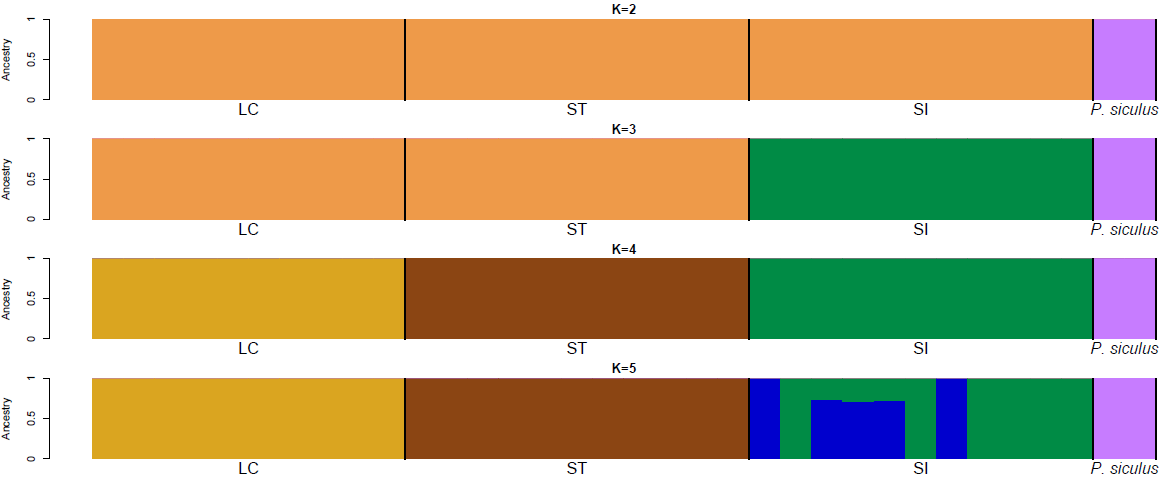


Fig. S6. Admixture analysis for 36 individuals from our three groups of interest, the Aeolian wall lizard from La Canna (LC) and Strombolicchio (ST), and the Sicilian wall lizard (SI), plus the outgroup *P. siculus*, for K between 2 and 5.


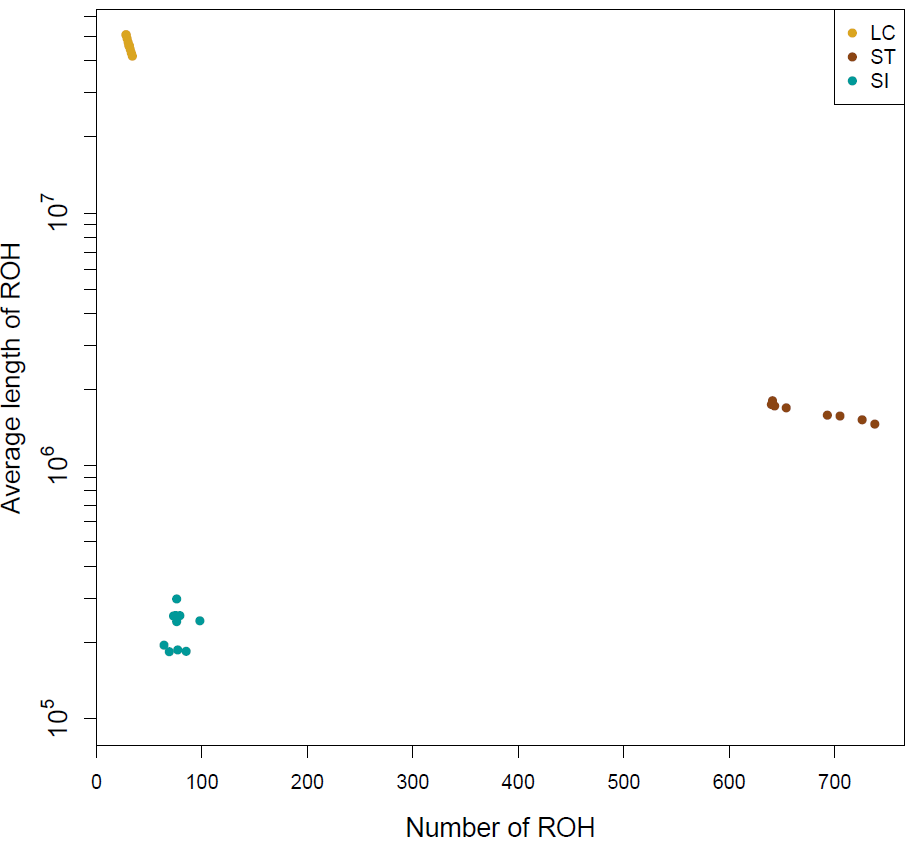


Fig. S7. Average length of ROH according to the number of ROH per individual, using a minimal ROH length of 100 kb, for Aeolian wall lizards from La Canna (LC) and Strombolicchio (ST), and for Sicilian wall lizards (SI).


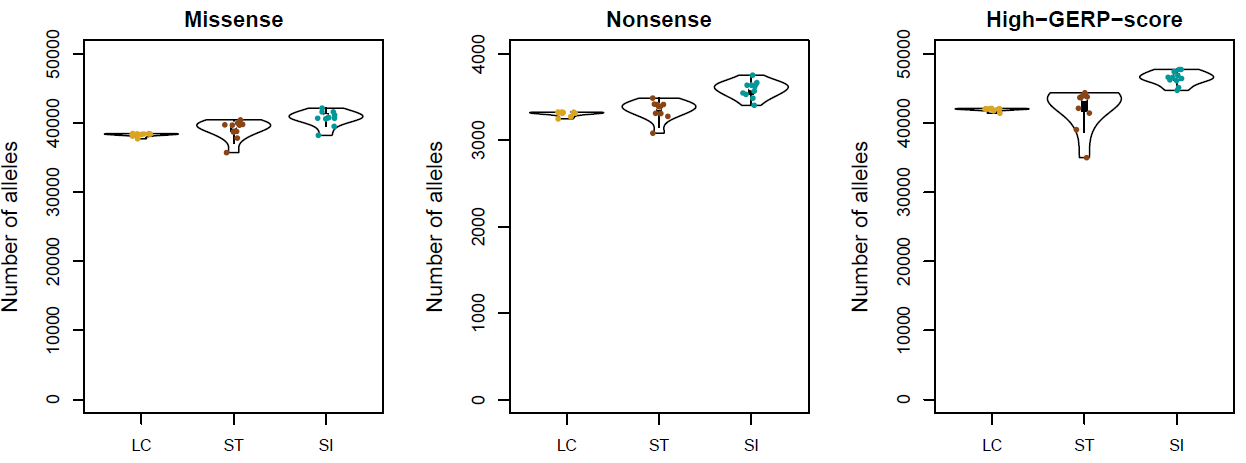


Fig. S8. Total count of putatively deleterious alleles present in each of the three groups of interest: the Aeolian wall lizard from La Canna (LC) and Strombolicchio (ST), and the Sicilian wall lizard (SI).


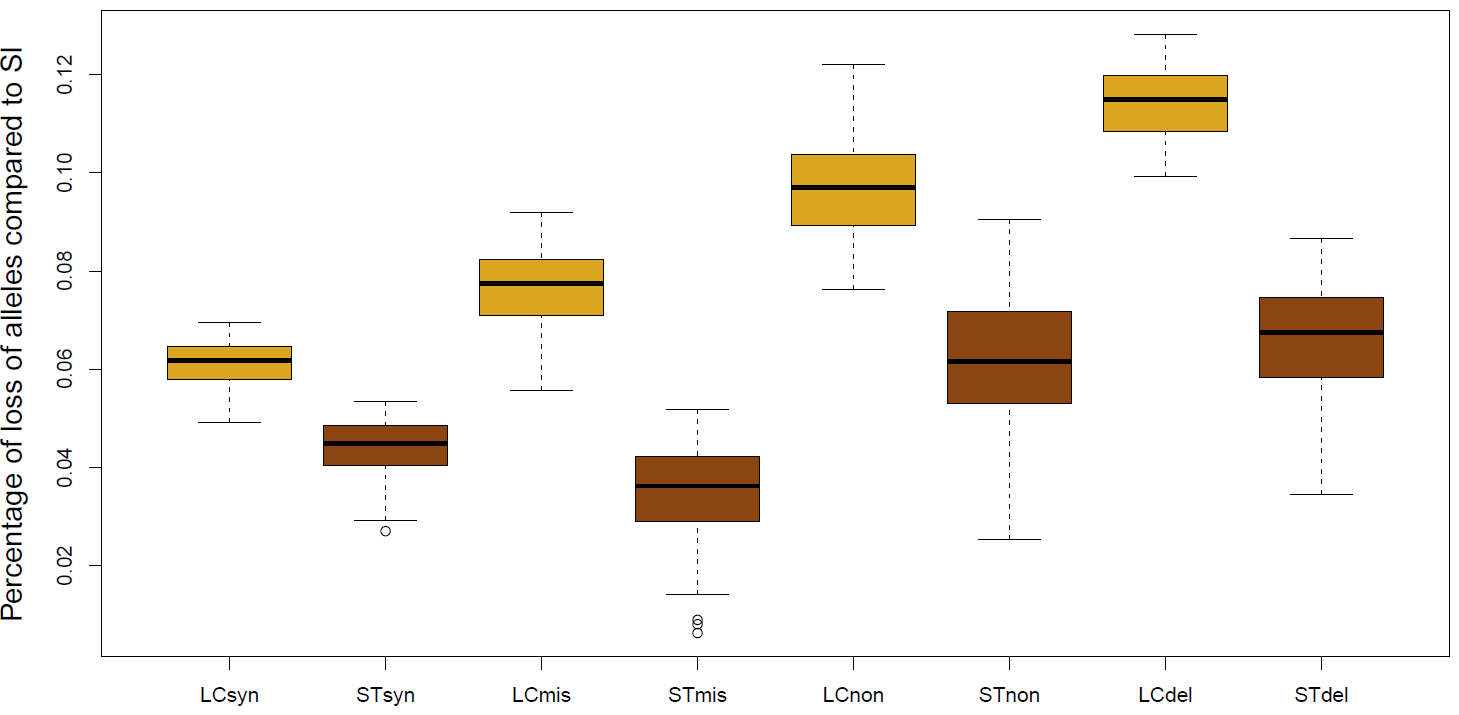


Fig. S9. Relative reduction of the number of alleles in small populations (Aeolian wall lizard from La Canna and Strombolicchio) compared to the Sicilian wall lizard (large population size), for near-neutral synonymous alleles (syn) and putatively deleterious alleles (mis: missense, non: nonsense, del: GERP score > 4).

Here, the individual allele counts have been corrected by the total number of alleles genotyped to account for differences in levels of missing data, that are particularly impactful when doing individual pairwise comparisons. We computed the ratio of the proportion of genotyped alleles between each possible pair of La Canna (LC) and Sicilian wall lizard individuals (yellow boxplots), and between each possible pair of Strombolicchio (ST) and Sicilian wall lizard individuals (brown boxplots).

**Fig. S10.** Distributions of GERP scores for the Aeolian wall lizard (LC and ST populations) and for the Sicilian wall lizard, with arithmetic mean, arithmetic mean for positive GERP scores, proportion of positive GERP scores, mean of GERP scores higher than 4 and proportion of sites with GERP>4.

Table S1. Details about the 32 individuals resequenced for the purpose of this study.

| **Sample ID** | **Species** | **Lineage** | **Locality** | **Latitude** | **Longitude** | **Sex** |
| --- | --- | --- | --- | --- | --- | --- |
| LC01 | *Podarcis raffonei* | ─ | La Canna | 38.345613 | 14.311661 | M |
| LC02 | *Podarcis raffonei* | ─ | La Canna | 38.345613 | 14.311661 | F |
| LC03 | *Podarcis raffonei* | ─ | La Canna | 38.345613 | 14.311661 | M |
| LC04 | *Podarcis raffonei* | ─ | La Canna | 38.345613 | 14.311661 | M |
| LC07 | *Podarcis raffonei* | ─ | La Canna | 38.345613 | 14.311661 | F |
| LC08 | *Podarcis raffonei* | ─ | La Canna | 38.345613 | 14.311661 | F |
| LC10 | *Podarcis raffonei* | ─ | La Canna | 38.345613 | 14.311661 | F |
| LC11 | *Podarcis raffonei* | ─ | La Canna | 38.345613 | 14.311661 | F |
| LC12 | *Podarcis raffonei* | ─ | La Canna | 38.345613 | 14.311661 | F |
| LC13 | *Podarcis raffonei* | ─ | La Canna | 38.345613 | 14.311661 | F |
| ST01 | *Podarcis raffonei* | ─ | Strombolicchio | 38.817154 | 15.251854 | F |
| ST02 | *Podarcis raffonei* | ─ | Strombolicchio | 38.817154 | 15.251854 | M |
| ST03 | *Podarcis raffonei* | ─ | Strombolicchio | 38.817154 | 15.251854 | F |
| ST04 | *Podarcis raffonei* | ─ | Strombolicchio | 38.817154 | 15.251854 | M |
| ST05 | *Podarcis raffonei* | ─ | Strombolicchio | 38.817154 | 15.251854 | F |
| DB25900 | *Podarcis raffonei* | ─ | Strombolicchio | 38.817154 | 15.251854 | F |
| DB26046 | *Podarcis raffonei* | ─ | Strombolicchio | 38.817154 | 15.251854 | F |
| DB26056 | *Podarcis raffonei* | ─ | Strombolicchio | 38.817154 | 15.251854 | F |
| DB26087 | *Podarcis raffonei* | ─ | Strombolicchio | 38.817154 | 15.251854 | M |
| DB26109 | *Podarcis raffonei* | ─ | Strombolicchio | 38.817154 | 15.251854 | M |
| DB23478 | *Podarcis waglerianus* | Western | Vita (TP) | 37.89153 | 12.80127 | M |
| DB23479 | *Podarcis waglerianus* | Western | Vita (TP) | 37.89153 | 12.80127 | F |
| DB23465 | *Podarcis waglerianus* | Western | Lago Preola e Gorghi Tondi (TP) | 37.60955 | 12.64847 | M |
| DB23461 | *Podarcis waglerianus* | Western | Lago Preola e Gorghi Tondi (TP) | 37.60955 | 12.64847 | F |
| DB23497 | *Podarcis waglerianus* | Western | Lago Preola e Gorghi Tondi (TP) | 37.60955 | 12.64847 | M |
| DB23481 | *Podarcis waglerianus* | Western | Lago Preola e Gorghi Tondi (TP) | 37.60955 | 12.64847 | F |
| DB23647 | *Podarcis waglerianus* | Eastern | Portopalo (SR) | 36.66816 | 15.09963 | M |
| DB23686 | *Podarcis waglerianus* | Eastern | Castagna (CT) | 37.26301 | 14.86462 | F |
| DB23687 | *Podarcis waglerianus* | Eastern | Castagna (CT) | 37.26301 | 14.86462 | M |
| DB23688 | *Podarcis waglerianus* | Eastern | Castagna (CT) | 37.26301 | 14.86462 | M |
| DB23668 | *Podarcis siculus* | Outgroup | Ispica (RG) | 36.76251 | 14.95547 | M |
| DB23669 | *Podarcis siculus* | Outgroup | Ispica (RG) | 36.76251 | 14.95547 | F |

Table S2. Summary of individual mapping statistics for the 38 *Podarcis* individuals mapped to the Aeolian wall lizard reference genome.

The percentage of callable bases was computed with GATK CallableLoci with a minimum base and mapping quality of 20 and a depth comprised between one third and three times the mean individual depth, as an estimate of the portion of the genome for which we can confidently call variants.

|  | Mean value |
| --- | --- |
| PERCENTAGE_READS_IN_PAIR_AFTER_TRIMMING (%) | 98 |
| PERCENTAGE_READS_MAPPED_IN_PAIRS (%) | 98 |
| MEAN_DEPTH_OF_COVERAGE | 16.1 |
| PERCENTAGE _COVERED_BASES (%) | 97 |
| PERCENTAGE _CALLABLE_BASES (%) | 83 |

Table S3. Genetic diversity statistics for the three groups of interest: the Aeolian wall lizard from La Canna (LC) and Strombolicchio (ST), and the Sicilian wall lizard (SI).

| **Population/**  **Species** | **Number of biallelic SNPs** | **Nucleotide diversity** | **Mean number of heterozygous sites**  **per individual** | **Expected**  **heterozygosity** | **Observed**  **heterozygosity** |
| --- | --- | --- | --- | --- | --- |
| LC | 10810 | 6.09E-06 | 3.29E-06 | 3.18E-06 | 3.35E-06 |
| ST | 335876 | 8.61E-05 | 1.35E-04 | 1.32E-04 | 1.35E-04 |
| SI | 17016396 | 2.83E-03 | 4.77E-03 | 5.06E-03 | 4.78E-03 |

Table S4. *Ne* estimates from different classes of ROH lengths corresponding to different coalescent times (in generations ago), for the Aeolian wall lizard from La Canna (LC) and Strombolicchio (ST).

| **_pop_ ^time (gen)^** | 4 | 9 | 18 | 36 | 179 |
| --- | --- | --- | --- | --- | --- |
| LC | 1 | 1 | 2 | 4 | 18 |
| ST | 5 | 6 | 8 | 14 | 60 |

Table S5. Individual counts of genetic load, for synonymous sites (near-neutral), and different classes of putatively deleterious mutations: missense, nonsense and high-GERP-score mutations. For each mutation type and each individual, we report the number of homozygous derived (der.hom) and heterozygous (het) genotypes and the number of derived alleles (nb.all).

|  |  | SYNONYMOUS SITES | | | MISSENSE SITES | | | NONSENSE SITES | | | HIGH-GERP-SCORE SITES | | |
| --- | --- | --- | --- | --- | --- | --- | --- | --- | --- | --- | --- | --- | --- |
| pop | ind | der.hom | het | nb.all | der.hom | het | nb.all | der.hom | het | nb.all | der.hom | het | nb.all |
| LC | LC01 | 41063 | 23 | 82149 | 19190 | 38 | 38418 | 1661 | 5 | 3327 | 21015 | 33 | 42063 |
| LC | LC02 | 41102 | 13 | 82217 | 19196 | 33 | 38425 | 1661 | 4 | 3326 | 21021 | 27 | 42069 |
| LC | LC03 | 41063 | 18 | 82144 | 19180 | 38 | 38398 | 1657 | 1 | 3315 | 21004 | 21 | 42029 |
| LC | LC04 | 40728 | 16 | 81472 | 19032 | 48 | 38112 | 1636 | 4 | 3276 | 20880 | 37 | 41797 |
| LC | LC07 | 40367 | 21 | 80755 | 18844 | 35 | 37723 | 1622 | 4 | 3248 | 20696 | 33 | 41425 |
| LC | LC08 | 41143 | 14 | 82300 | 19223 | 33 | 38479 | 1662 | 3 | 3327 | 21044 | 18 | 42106 |
| LC | LC10 | 41023 | 17 | 82063 | 19157 | 35 | 38349 | 1654 | 4 | 3312 | 20988 | 32 | 42008 |
| LC | LC11 | 41085 | 15 | 82185 | 19186 | 37 | 38409 | 1658 | 2 | 3318 | 21004 | 28 | 42036 |
| LC | LC12 | 41121 | 15 | 82257 | 19214 | 36 | 38464 | 1660 | 1 | 3321 | 21033 | 26 | 42092 |
| LC | LC13 | 40987 | 15 | 81989 | 19162 | 47 | 38371 | 1656 | 3 | 3315 | 21002 | 30 | 42034 |
| ST | DB25900 | 41121 | 1018 | 83260 | 19588 | 705 | 39881 | 1682 | 50 | 3414 | 21606 | 712 | 43924 |
| ST | DB26046 | 40904 | 864 | 82672 | 19520 | 660 | 39700 | 1665 | 63 | 3393 | 21476 | 740 | 43692 |
| ST | DB26056 | 41003 | 892 | 82898 | 19567 | 670 | 39804 | 1668 | 55 | 3391 | 21549 | 672 | 43770 |
| ST | DB26087 | 40988 | 831 | 82807 | 19593 | 554 | 39740 | 1688 | 39 | 3415 | 21539 | 711 | 43789 |
| ST | DB26109 | 40930 | 715 | 82575 | 19593 | 509 | 39695 | 1667 | 58 | 3392 | 21510 | 667 | 43687 |
| ST | SRR14009404 | 41308 | 932 | 83548 | 19695 | 1062 | 40452 | 1690 | 109 | 3489 | 21599 | 1184 | 44382 |
| ST | ST01 | 39990 | 891 | 80871 | 19046 | 616 | 38708 | 1630 | 53 | 3313 | 20698 | 739 | 42135 |
| ST | ST02 | 41297 | 832 | 83426 | 19672 | 648 | 39992 | 1678 | 63 | 3419 | 21690 | 679 | 44059 |
| ST | ST03 | 36725 | 836 | 74286 | 17526 | 661 | 35713 | 1513 | 57 | 3083 | 17181 | 620 | 34982 |
| ST | ST04 | 38898 | 917 | 78713 | 18551 | 721 | 37823 | 1606 | 65 | 3277 | 19130 | 782 | 39042 |
| ST | ST05 | 39997 | 962 | 80956 | 19012 | 760 | 38784 | 1620 | 70 | 3310 | 20343 | 755 | 41441 |
| SI | DB23461 | 20134 | 45308 | 85576 | 9600 | 21494 | 40694 | 843 | 1884 | 3570 | 9421 | 27399 | 46241 |
| SI | DB23465 | 19967 | 46277 | 86211 | 9344 | 22007 | 40695 | 857 | 1924 | 3638 | 9340 | 27764 | 46444 |
| SI | DB23478 | 19773 | 47396 | 86942 | 9224 | 22704 | 41152 | 818 | 1985 | 3621 | 9129 | 28619 | 46877 |
| SI | DB23479 | 19427 | 44678 | 83532 | 9196 | 21133 | 39525 | 843 | 1802 | 3488 | 9350 | 26434 | 45134 |
| SI | DB23481 | 20166 | 47317 | 87649 | 9556 | 22498 | 41610 | 838 | 1992 | 3668 | 9369 | 28693 | 47431 |
| SI | DB23497 | 19424 | 47032 | 85880 | 9073 | 22464 | 40610 | 820 | 1892 | 3532 | 9069 | 27998 | 46136 |
| SI | DB23647 | 23446 | 35018 | 81910 | 10731 | 16766 | 38228 | 962 | 1482 | 3406 | 11728 | 21288 | 44744 |
| SI | DB23686 | 24338 | 38072 | 86748 | 11297 | 19016 | 41610 | 982 | 1688 | 3652 | 12100 | 23545 | 47745 |
| SI | DB23687 | 24608 | 36557 | 85773 | 11280 | 18227 | 40787 | 1011 | 1616 | 3638 | 12147 | 22334 | 46628 |
| SI | DB23688 | 24526 | 36284 | 85336 | 11212 | 18360 | 40784 | 986 | 1576 | 3548 | 12186 | 22235 | 46607 |
| SI | SRR14009413 | 21316 | 45152 | 87784 | 9936 | 22291 | 42163 | 894 | 1967 | 3755 | 10118 | 27512 | 47748 |

Table S6. Individual counts of high-GERP-score mutations identified as missense and nonsense by SnpEff, with the number of homozygous derived (der.hom) and heterozygous (het) genotypes and the number of derived alleles (nb.all).

|  |  | HIGH-GERP-SCORE MISSENSE SITES | | | HIGH-GERP-SCORE NONSENSE SITES | | |
| --- | --- | --- | --- | --- | --- | --- | --- |
| pop | ind | der.hom | het | nb.all | der.hom | het | nb.all |
| LC | LC01 | 2350 | 10 | 4710 | 235 | 2 | 472 |
| LC | LC02 | 2353 | 9 | 4715 | 234 | 2 | 470 |
| LC | LC03 | 2357 | 12 | 4726 | 235 | 0 | 470 |
| LC | LC04 | 2337 | 13 | 4687 | 229 | 2 | 460 |
| LC | LC07 | 2316 | 15 | 4647 | 230 | 1 | 461 |
| LC | LC08 | 2361 | 7 | 4729 | 235 | 0 | 470 |
| LC | LC10 | 2350 | 14 | 4714 | 234 | 1 | 469 |
| LC | LC11 | 2355 | 7 | 4717 | 235 | 1 | 471 |
| LC | LC12 | 2358 | 10 | 4726 | 235 | 0 | 470 |
| LC | LC13 | 2353 | 13 | 4719 | 234 | 1 | 469 |
| ST | DB25900 | 2378 | 128 | 4884 | 221 | 10 | 452 |
| ST | DB26046 | 2370 | 135 | 4875 | 223 | 9 | 455 |
| ST | DB26056 | 2369 | 106 | 4844 | 218 | 11 | 447 |
| ST | DB26087 | 2363 | 121 | 4847 | 222 | 9 | 453 |
| ST | DB26109 | 2375 | 117 | 4867 | 218 | 11 | 447 |
| ST | SRR14009404 | 2395 | 254 | 5044 | 226 | 33 | 485 |
| ST | ST01 | 2302 | 124 | 4728 | 219 | 11 | 449 |
| ST | ST02 | 2392 | 109 | 4893 | 225 | 10 | 460 |
| ST | ST03 | 2008 | 113 | 4129 | 191 | 11 | 393 |
| ST | ST04 | 2178 | 149 | 4505 | 201 | 12 | 414 |
| ST | ST05 | 2267 | 149 | 4683 | 212 | 13 | 437 |
| SI | DB23461 | 983 | 2767 | 4733 | 106 | 301 | 513 |
| SI | DB23465 | 944 | 2838 | 4726 | 107 | 280 | 494 |
| SI | DB23478 | 935 | 2917 | 4787 | 96 | 285 | 477 |
| SI | DB23479 | 960 | 2750 | 4670 | 118 | 281 | 517 |
| SI | DB23481 | 972 | 2873 | 4817 | 101 | 322 | 524 |
| SI | DB23497 | 969 | 2801 | 4739 | 105 | 275 | 485 |
| SI | DB23647 | 1123 | 2217 | 4463 | 140 | 218 | 498 |
| SI | DB23686 | 1198 | 2535 | 4931 | 129 | 256 | 514 |
| SI | DB23687 | 1203 | 2339 | 4745 | 132 | 251 | 515 |
| SI | DB23688 | 1240 | 2361 | 4841 | 144 | 230 | 518 |
| SI | SRR14009413 | 1048 | 2908 | 5004 | 100 | 328 | 528 |

Table S7. Mean number of alleles at high frequency (frequency of the derived allele higher than 0.9) in the three groups of interest: the Aeolian wall lizard from La Canna (LC) and Strombolicchio (ST), and the Sicilian wall lizard (SI).

|  | **LC** | **ST** | **SI** |
| --- | --- | --- | --- |
| Synonymous | 41192 | 40849 | 8158 |
| Missense | 19231 | 19357 | 3936 |
| Nonsense | 1663 | 1653 | 348 |
| High-GERP-score | 21056 | 21297 | 3407 |

Table S8. Mean individual number of alleles for near-neutral alleles (synonymous) and putatively deleterious alleles (missense, nonsense and high-GERP-score) for each group: the Aeolian wall lizard from La Canna (LC) and Strombolicchio (ST), and the Sicilian wall lizard (SI). Numbers inside brackets indicate the percentage of differences compared to the Sicilian wall lizard (SI), which represents our control group with large population size.

|  | **LC** | **ST** | **SI** |
| --- | --- | --- | --- |
| Synonymous | 81953 (-4.4%) | 81456 (-5.0%) | 85758 |
| Missense | 38315 (-5.9%) | 39117 (-3.9%) | 40714 |
| Nonsense | 3309 (-7.9%) | 3354 (-6.6%) | 3592 |
| High-GERP-score | 41966 (-9.8%) | 42264 (-9.2%) | 46521 |

**References**

Yang W, Feiner N, Pinho C, While GM, Kaliontzopoulou A, Harris DJ, *et al.* (2021). Extensive introgression and mosaic genomes of Mediterranean endemic lizards. *Nat Commun* **12**: 2762.
